# Current knowledge of large vascular occlusion due to intracranial atherosclerosis: focusing on early diagnosis

**DOI:** 10.1186/s41016-020-00213-1

**Published:** 2020-10-01

**Authors:** He Li, Peng Liu, Pei Liu, Weilong Hua, Wenjin Yang, Yongxin Zhang, Lei Zhang, Pengfei Xing, Zifu Li, Yongwei Zhang, Bo Hong, Pengfei Yang, Jianmin Liu

**Affiliations:** 1 Department of Neurosurgery, Changhai Hospital, Changhai Road Num. 168, Shanghai, 200433 China; 2 Stroke Center, Changhai Hospital, Changhai Road Num. 168, Shanghai, 200433 China

**Keywords:** Acute ischemic stroke, AIS, Intracranial atherosclerosis, ICAS, Large vascular occlusion, LVO, Diagnosis, ICAS-LVO

## Abstract

Intracranial atherosclerosis (ICAS)-related large vascular occlusion (LVO) was an intractable subtype of acute ischemic stroke (AIS), which always needed rescue angioplasty and stenting and complicated the procedure of endovascular recanalization. Diagnosing ICAS-LVO accurately and early was helpful for both clinical treatment and trials. Digital subtraction angiography (DSA) was unable to provide an early and rapid diagnosis of ICAS-LVO based on current studies. A variety of pre-DSA methods had been used to distinguish ICAS-LVO with other subtypes of ischemic stroke, such as medical histories, clinical presentations, computed tomography or angiography (CT/CTA), and magnetic resonance imaging (MRI/MRA). This article briefly reviewed the status quo of the diagnosis and treatment of ICAS-LVO and summarized early diagnostic methods of ICAS-LVO from different aspects.

## Background

Early in 1993, the ischemic stroke had been classified into five subtypes by Trial of Org 10172 in Acute Stroke Treatment (TOAST), in which large artery atherosclerosis (LAA) was regarded as a specific etiology of acute ischemic stroke AIS [1]. With the development of technology, endovascular treatment, including mechanical thrombectomy, has become the first-line therapy for AIS with large vessel occlusion, which requires clinicians to identify the etiology of AIS more accurately to provide an optimal treatment [2, 3].

Intracranial atherosclerosis (ICAS)-related large vascular occlusion (LVO) is a specific type of LAA which happens in intracranial cerebral arteries. The diagnosis and treatment of ICAS-LVO are more complicated than extracranial atherosclerotic LVO because of its unique position and anatomical features. Previous studies demonstrated that ICAS-LVO was more prevalent in the Asian population [4]. The strategies for treating cardioembolic stroke and ICAS-LVO are always not the same. Atherosclerotic stenosis usually needs to be solved by angioplasty with or without stent implement to recanalize the vessel fully. Further treatment was sometimes necessary to prohibit in situ thrombosis at the site of stenosis [5–7]. Rescue treatment is a complicated and time-consuming process that requires skilled operators and specific endovascular apparatus. Thus, diagnosing ICAS-LVO early and accurately becomes particularly important. However, methods based on digital subtraction angiography (DSA) cannot always provide an early diagnosis because it is an intra-operative diagnostic method. In this review, we pointed out the necessity of early diagnosis of ICAS-LVO. The current methods that can provide valuable information for the diagnosis of ICAS-LVO, including medical histories, clinical presentations, multi-model computed tomography (CT), and multi-model magnetic resonance imaging (MRI), were summarized.

## Status quo of the diagnosis and treatment of ICAS-LVO

### The diagnosis of ICAS-LVO

Once the patient is admitted to the hospital or even earlier, the medical histories are acquired from patients or the patient’s family. The symptoms and physical signs are also detailly recorded [8]. The National Institutes of Health Stroke Scale (NIHSS) was measured by experienced attending doctors. Non-contrast CT is then performed to exclude hemorrhage, and CT angiography (CTA) with CT perfusion-weighted imaging (CTP), or MR angiography (MRA) with MR perfusion-weighted imaging (MRP) are performed to acquire vascular and perfusion conditions and information about core infarction. However, these works were only able to confirm the diagnosis of AIS but not ICAS-LVO. The determinate diagnosis of ICAS-LVO is currently based on the DSA image during the endovascular procedure.

DSA is the golden standard for the diagnosis of AIS and ICAS-LVO. Physicians usually use residual stenosis of a cerebral artery after the first-pass thrombectomy as a golden criterium to diagnose ICAS-LVO. Some studies consider occlusions with remaining stenosis ≥ 50% after the first-pass thrombectomy as ICAS-LVO [9, 10]. The cutoff value for diagnosing ICAS-LVO is 70% residual stenosis in some other researches [11–13]. If the residual stenosis does not meet the criteria of ICAS-LVO, additional criteria will take effect [14–16]. For instance, if the stenosis is less than 50%, but its distal blood flow is impaired, or it tends to re-occlude, it will be considered an ICAS-LVO. As we can see, the diagnostic criteria of ICAS-LVO based on residual stenosis are ununified, which might lead to less accurate diagnosis and improper treatment. Thus, the criteria based on DSA should be unified, and other indicators of ICAS-LVO are required to assist the determinate diagnosis. For instance, the status of collateral circulation may also help verify the diagnosis of ICAS-LVO. The chronic stenosis of intracranial arteries may lead to a compensatory adjustment in the brain, such as the neovascularization and the open of collateral arteries [17, 18]. Recent studies showed that the collateral circulation was better in patients with ICAS-LVO than that without chronic stenosis, which means a higher grade of collateral circulation could predict ICAS-LVO [17, 19]. Another study by Baek and colleagues demonstrated the significance of occlusion type in early diagnosis of ICAS-LVO by DSA. In brief, they regarded occlusions at bifurcations or with invisible distal branches or bifurcations as branching-type occlusions and they considered occlusions at major arteries with visible distal branches or bifurcations as truncal-type occlusions [20]. Predicting the refractory occlusions is the original purpose of this method. Further studies confirmed the efficacy of truncal-type occlusion in predicting ICAS-LVO, and the diagnosis based on occlusion type had been used in several clinical researches [7, 21]. A novel intra-operative sign of ICAS-LVO called the “microcatheter first-pass effect” was identified recently. When the operator withdraws the microcatheter after it has been navigated across the occlusion, if a blood flow presented in the distal artery beyond the occlusion, the “microcatheter first-pass effect” is positive [22]. Based on the results from 61 patients with ICAS, both sensitivity and specificity of the “microcatheter first-pass effect” in diagnosing ICAS related AIS were nearly 90%. So, this sign is a promising indicator of ICAS after being confirmed by a study with a larger scale.

In clinical practice, diagnosing ICAS-LVO by DSA usually relies on the experience of physicians. Some other experiential indicators are often used to help diagnose ICAS-LVO, such as the tortuosity of the occluded artery and the condition of the microcatheter during the procedure [23]. Further experiments are necessary to confirm their efficacy.

### Treatment of ICAS-LVO at the acute stage

After non-contrast CT scanning and CTA with CTP (or MRA with MRP), intravenous alteplase infusion is immediately administrated if the patient was eligible [8]. Mechanical thrombectomy (MT) is always regarded as the most effective reperfusion therapy for AIS within the time window. Studies in these 5 years had proved that MT with intravenous alteplase was superior to intravenous alteplase alone, which increased the successful reperfusion (mTICI 2b-3) rate to 70-80% and the rate of a good outcome to 40-50% [2]. However, these results were based on the overall population of AIS but not on the population of ICAS-LVO. A series of studies focused on ICAS-LVO indicated that using endovascular therapy to treat ICAS-LVO was also applicable. In many studies, endovascular treatments, including stent-retriever, aspiration thrombectomy, and angioplasty, were used as primary therapy, and infusion of glycoprotein IIB/IIIA receptor inhibitor and stent implantation were performed as rescue therapy [5, 24]. With the help of rescue therapies, the safety and efficacy of endovascular treatment in dealing with ICAS-LVO were comparable or even better than those in embolic cases. Nevertheless, many details remain undetermined in the endovascular treatment of ICAS-LVO. Firstly, the optimal primary therapy for ICAS-LVO remains unknown. It is gradually accepted that MT is the first-line endovascular therapy in treating LVO, and the efficacy and safety of stent retriever and aspiration thrombectomy were comparable [25, 26]. However, whether these notions are applicable in dealing with ICAS-LVO is unknown. A study by Kang et al. illustrated that stent retriever was superior to aspiration from different aspects [27]. According to their results, the successful rate of stent retriever as the primary therapy was higher than aspiration. The procedure time, which might influence clinical outcome, was shorter in the stent-retriever group. Another research by Yang and colleagues compared the efficacy and safety between angioplasty with stenting and MT as the primary therapy for ICAS-LVO [28]. Their results indicated that first-line angioplasty with stenting was superior to MT from the aspects of 90-day outcome and rate of asymptomatic intracranial hemorrhage. These initial results were meaningful for further investigation of the first-line endovascular therapy of ICAS-LVO. Secondly, the rescue strategy for failed MT in dealing with ICAS-LVO was undetermined. It had been illustrated that rescue therapies were often required for full recanalization of ICAS-LVO, which might increase the complexity of the procedure and prolong the procedure time [5, 29]. However, there is a lack of study focusing on the optimal rescue strategy for ICAS-LVO, which is a pending issue in clinical practice.

### The necessity of early diagnosis of ICAS-LVO

**Fig. 1:**
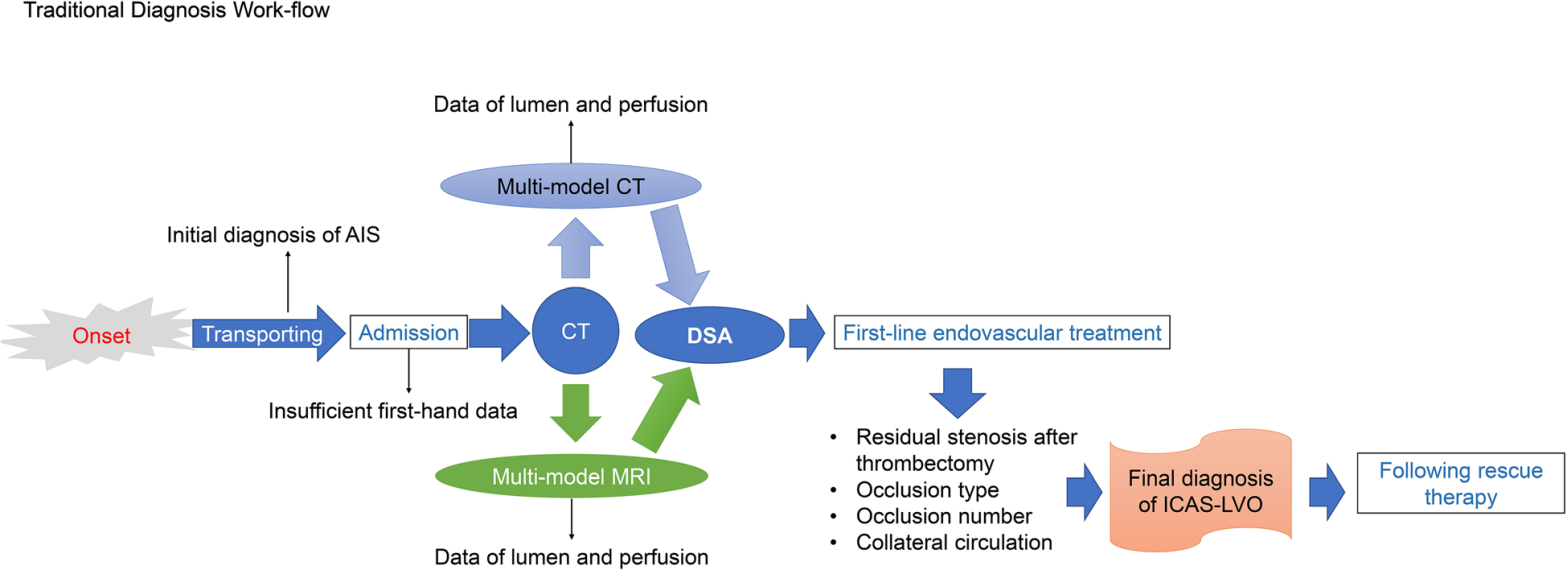
Traditional diagnosis work-flow of ICAS-LVO

Based on current knowledge, the treatment of ICAS-LVO always required rescue therapy and longer procedure time [5, 24]. This requires clinicians to make an early and accurate diagnosis so that the operators can acquire sufficient time and information to formulate a beneficial treatment strategy and prepare for the endovascular treatment. However, the diagnosis of ICAS-LVO is still relying on DSA images after the first-line thrombectomy, which is not able to provide a diagnosis early and accurately enough. The contradiction between the requirement of precise treatment and the hysteretic diagnosis needs to be settled by novel diagnostic strategies (Fig. 1). Besides, the optimal primary and rescue strategy for ICAS-LVO is still undetermined. One reason for this circumstance is that the diagnosis of ICAS-LVO based on DSA makes it hard to conduct prospective research focusing on ICAS-LVO. Moreover, the diagnosis is also less reliable based on ununified DSA criteria. Thus, other methods are needed to improve the diagnostic accuracy and provide early diagnosis of ICAS-LVO.

## Potential methods of diagnosing ICAS-LVO

### Medical histories and clinical presentations

Medical histories and clinical presentations are the first-hand data for clinicians. Some medical histories can be valuable to the rapid diagnosis of ICAS-LVO related AIS. A patient who suffers intermittent claudication or symptoms related to transient ischemic attacks (TIAs) repeatedly on the same side before AIS should be suspected of having ICAS-LVO [1]. Other clinical histories or risk factors, including hypertension, diabetes mellitus, dyslipidemia, and smoking history, are also more prevalent in patients with ICAS-LVO than those with embolic stroke [5]. History of previous or continuous atrial fibrillation is a negative indicator of ICAS-LVO.

The severity of symptoms in patients with ICAS-LVO is usually less than that in patients with cardioembolic AIS, manifesting as lower NIHSS in the ICAS-LVO group [5, 24]. Some clinical presentations can provide clues to the diagnosis of ICAS-LVO. Carotid bruit is a classical sign of extracranial carotid artery stenosis, which reveals underlying extracranial atherosclerosis and a poor vascular condition of the patient [30, 31]. A study by Brutto et al. illustrated the relationship between increased brachial pulse pressure (PP) and carotid siphon calcification, a sign of ICAS [32]. Although carotid bruit and brachial PP are not direct indicators of ICAS-LVO, they can be valuable for the early diagnosis of ICAS-LVO. However, no research has been published, confirming the diagnostic significance of these medical histories and clinical presentations.

The leading concern of these indicators is the accuracy of data acquired from amateurs. Attending doctors should prudently identify the valuable information from redundant complaints of the patients and their families.

### Multi-model CT

CT is now routinely performed when diagnosing AIS. A plain CT scan can present the infarcted territory and provide some clues of the etiology of AIS. High-density plaques in the CT scans can be observed in patients with symptomatic ICAS, which may also be observed in patient ICAS-LVO related AIS [33, 34]. This sign usually indicates the calcification of atherosclerotic plaque. However, in some other cases, high-density sign in plain CT scan also indicates calcified or erythrocyte-enriched thrombi, which are evidence for cardioembolic AIS [35, 36]. Thus, it may be confused to regard high-density sign as an indicator of ICAS-LVO. The Alberta Stroke Program Early CT Score (ASPECTS), an indicator of infarcted size based on plain CT scan, maybe a potential indicator of ICAS-LVO. According to several studies, the ASPECTS is always relatively higher in the ICAS-LVO group than in the embolic-LVO group [37, 38]. Similarly, computed tomography perfusion imaging (CTP) also shows differences in the core infarcted area between ICAS-LVO and embolic-LVO [38]. Currently, no data illustrated the diagnostic value of the differences of ASPECTS and CTP core infarction between the ICAS-LVO group and the embolic-LVO group. However, several studies have demonstrated the value of volume and location of core infarction based on DWI in diagnosing ICAS-LVO [39, 40]. Similar to DWI, CTP and ASPECTS can also manifest the location and volume of core infarction, and their diagnostic value to ICAS-LVO deserves further investigation.

CTA can provide more information about vascular lumen conditions, and it is also routinely performed in many centers along with plain CT scan [8]. CTA is minimally invasive and costs less time than MRA [41]. Many studies demonstrated the efficacy of CTA in diagnosing ICAS and acute LVO-related AIS, and its sensitivity was promising in referencing the DSA [42–44]. However, the efficacy of CTA in distinguishing ICAS-LVO-related AIS from other etiologies of stroke is unknown. The most significant barrier for CTA to distinguish ICAS-LVO is that CTA is not able to find significant differences between ICAS-LVO and embolic-LVO at the occlusion site based on current technology. Operators can only speculate whether the occlusion is due to ICAS-LVO and prepare for the intervention by estimating the vascular condition near the occlusion site through CTA image [42]. For this reason, further studies were conducted to identify other valuable characteristics of CTA images in diagnosing ICAS-LVO. Guglielmi et al. showed that the ratio of good collateral condition was higher in the ICAS-LVO group than in the cardioembolic group, which indicated that CTA might be able to distinguish ICAS-LVO through collateral grade before DSA was performed [45]. Another study by Rebello et al. demonstrated that patients with ICAS-LVO predominantly had higher grade (grades 2-4) of collateral circulation based on CTA image, and the specificity of grades 2-4 collateral circulation in diagnosing ICAS-LVO was nearly 90% [46]. Some studies demonstrated the significance of occlusions’ features based on CTA in the early diagnosis of ICAS-LVO. A study by Chen and colleagues pointed out the value of multi-segment clot (MSC) sign on CTA in identifying the etiology of acute LVO [47]. MSC sign is defined as more than one occlusive lesion on CTA. The occurrence of MSC sign was significantly higher in cardioembolic and undetermined AIS than in the LAA-AIS group, so it could be considered an exclusive criterion for ICAS-LVO. A series of studies by Baek et al. founded the value of occlusion type based on CTA in judging stroke etiology. Similar to DSA, the truncal-type occlusion was defined as occlusion in which the bifurcation site could be clearly observed, and branching type was assigned as occlusion in which the bifurcation site was not able to be observed. They demonstrated that CTA was also available in judging occlusion type, based on which clinicians could differentiate ICAS positive LVO and ICAS negative LVO [21]. Besides occlusion type, the location of occlusions also has potential in predicting in ICAS-LVO. Many studies reported that ICAS lesions tended to be located at the proximal M1 segment for anterior circulation and the proximal or middle basilar artery (BA) segment for posterior circulation. Much less ICAS lesions were located at distal segments, such as M1 bifurcation, M2, BA tip, and posterior cerebral artery [48, 49]. These data indicated that the occlusions located at segments with a higher risk of ICAS being much likely to be ICAS-LVOs. Thus, the location of occlusion might be a potential predictor of ICAS-LVO. It is necessary to define the scope of every segment of cerebral vessels accurately to identify the correlation between the location of occlusions and the diagnosis of ICAS-LVO. Furthermore, some other indicators for ICAS-LVO may have great potential but still lack of evidence. Based on our clinical experience and previous studies, ICAS always causes a length of stenosis of intracranial arteries, and ICAS-LVO usually accompanies the stenosis of the artery proximal to the occlusion site [50, 51]. This means that a segment of progressive narrowing artery from distal to proximal to the occlusion site can be observed by CTA in patients with ICAS-LVO, which could be a candidate for the diagnostic criteria of ICAS-LVO and deserved further investigation [52].

In summary, CTA is an efficient, minimally invasive, and informative diagnostic method which can provide us information about the location of the occlusion, the condition of entire cerebral vessels and collateral circulation, and the occlusion types. The application of CTA may be limited currently because of the necessity of contrast agents and radiation (Table 1). Multi-model CT scans, especially CTA, have great potential in predicting ICAS-LVO related AIS.

**Table 1:** Comparison of three common imaging methods in diagnosing ICAS-LVO

	Comparing with DSA	Comparing with multi-model CT	Comparing with multi-model MRI
DSA		Strength: 1. Higher accuracy	Strength: 1. Higher accuracy
		Weakness: 1. Later than treatment 2. Treatment dependent	Weakness: 1. Later than treatment 2. Treatment dependent
Mult-model CT	Strength: 1. Early diagnosis 2. Treatment independent		Strength: 1. Rapid 2. Lower hardware requirement 3. Usually routinely performed
	Weakness: 1. Less accuracy (based on current knowledge)		Weakness: 1. Less informative about the occlusion site 2. Invasive process 3. Radiation
Multi-model MRI	Strength: 1. Early diagnosis 2. Treatment independent	Strength: 1. More informative about the occlusion site 2. Usually noninvasive 3. Without radiation	
	Weakness: 1. Time-consuming 2. Higher equipment requirement 3. Unsuitable for patients with magnetic sensitive implant	Weakness: 1. Time-consuming 2. Higher hardware requirement 3. Unsuitable for patients with magnetic sensitive implant 4. Require strict immobilization of the patient	

### Multi-model MRI

MRI is another common method in diagnosing AIS. T2-weighted MRI is more sensitive than plain CT scan in diagnosing cerebral infarction, and diffusion-weighted imaging (DWI) can identify hyperacute ischemic stroke [53]. A study by Kim et al. demonstrated the value of the infarct volume estimated by DWI in diagnosing ICAS-LVO [39]. They found that the median infarct volume in DWI was significantly higher in the cardioembolic-LVO group than in the ICAS-LVO group. However, it is difficult to find a threshold of infarct volume to diagnose ICAS-LVO because of the heterogeneity of patients. Another study by Zhang et al. demonstrated the significance of core infarction location based on DWI in predicting ICAS-LVO [40]. Their results indicated that the core infarction induced by ICAS-LVO always involves the deep part of the brain, such as in the basal ganglia or semiovoid region. The sensitivity and specificity of the core infarction location were 93.3% and 87.5% respectively in predicting ICAS-LVO. Investigation with a larger scale is needed to confirm their finding.

MRA based on time of flight (TOF) or contrasted enhancement technique is performed in some stroke centers to get more information about the lumen to diagnose ICAS-LVO [41, 54, 55]. Similar to CTA, MRA was only able to provide some indirect evidence for ICAS-LVO, such as the occlusion type and collateral circulation, but could not directly distinguish ICAS-LVO from other etiologies of stroke by the image characteristics at the occlusion site. Besides, the diagnostic efficiency of MRA alone (based on 3D-TOF performed with 1.5-T MRI apparatus) is also inferior to CTA in diagnosing ICAS-related stenosis, which meant that CTA might be more useful than MRA in distinguishing ICAS-LVO [56].

Vessel wall MRI (vwMRI) is a novel technology that can better manifest the atherosclerotic plaque and define the etiology of AIS [57]. Just as its name implies, vwMRI is a method focusing on the lesion of the vessel wall, which is different from “lumen imaging” methods such as CTA and MRA. VwMRI takes advantage of fast-spin echo and double-inverse recovery technology to suppress the signal of blood flow and present a “black blood” image showing the morphology of atherosclerotic plaque [58]. Its shortcoming of low spatial resolution could be compensated by three-dimensional techniques, such as VISTA (Philips Healthcare, the Netherlands), SPACE (Siemens Healthcare, Germany), and CUBE (GE Healthcare, USA) [59, 60]. VwMRI has long been used in the diagnosis of chronic symptomatic or asymptomatic ICAS. The consistency is excellent between vwMRI and DSA in estimating the degree of stenosis in MCA, and the sensitivity of vwMRI is higher than CTA and MRA (3D TOF) [61, 62]. The excellent efficacy of vwMRI also primarily presents in diagnosing ICAS-LVO. Kim and colleagues observed decreased blood lumen area of patients with ICAS-LVO on vwMRI [63]. Ryoo et al. recognized the vessel wall enhancement on T1-weighted post-contrast vwMRI of patients with ICAS-LVO [64]. Hui and colleagues further identified several essential characteristics of vwMRI in patients with ICAS-LVO, such as hyperintense on post-contrast T1 vwMRI of the distal lumen or “white snake sign,” narrowed of both inner and outer wall, and concentric enhancement and thickening of the wall [65]. These characteristics deserve to be confirmed by investigations with larger scales. Another sign called artery remodeling also became applicable in diagnosing ICAS-LVO with the help of vwMRI. The conception of vascular remodeling is firstly raised by cardiologists, and it is introduced to the cerebral vascular system because similar conditions are also presented in ICAS. There are two patterns of remodeling, including positive remodeling (PR) and negative remodeling (NR) [66]. PR represented that the current outer diameter of an artery is larger than its original outer diameter (remodeling ratio > 1.05), and PR represented arteries with smaller outer diameter than its original outer diameter (remodeling ratio < 0.95). Several researches demonstrated that PR at the stenosis in ICAS was related to the risk of ischemic stroke [67]. Thus, PR at the occlusion site might be an indicator of the etiology of ICAS-LVO, which needs further confirmation.

Other sequences of MRI might also contribute to the early diagnosis of ICAS-LVO. Susceptibility vessel sign (SVS) based on susceptibility-weighted imaging (SWI) is a parameter in judging the etiology of AIS [68]. SVS refers to the hypointensity inside arteries accompanied by a blooming artifact, the width of which is significantly larger than the real diameter of the artery. This sign will lead to the overestimation of the width of the artery. The predominant component of cardiogenic thrombus is red blood cells, which can cause significant SVS in patients with cardioembolic AIS. Zhang et al. found that the sensitivity and specificity of the overestimation ratio of SVS > 2.003 in identifying cardioembolic LVO was 97.1% and 91.3%, respectively [68]. Contrarily, the sensitivity and specificity of the overestimation ratio of SVS ≤ 2.003 was 91.3% and 97.1% respectively in diagnosing CAS-LVO. Another study by Yamamoto et al. further indicated that 2-layered SVS, a specific SVS with a low-intensity core surrounded by a higher intensity layer, had higher specificity (97.1%) than simple SVS in identifying cardioembolic LVO [69]. Thus, SVS and 2-layered SVS can provide reliable evidence to rule-out the diagnosis of cardioembolic LVO, which indirectly improved the accuracy of the diagnosis of ICAS-LVO.

The diagnostic methods based on multi-model MRI are more informative than multi-model CT. However, the leading limitation of MRI is its long scan time. Based on 3.0-T field strength apparatus, the whole series of MRI sequences including T1-, T2-weighted sequences, 3D TOF MRA sequences, and vwMRI sequences costs more than 30 min at least, which is much longer than CTA and delays the endovascular treatment [57, 60] (Table 1). It is also hard to keep the unconscious patient strictly still in the instrument for such a long time. Some stroke centers even do not have a 3.0-T field strength MRI yet. Thus, based on current technical consideration, multi-model MRI may not be as applicable as multi-model CT. The diagnostic value of MRI will further increase with the availability of equipment requiring shorter scanning time and higher resolution.

### Other indicators contribute to the diagnosis of ICAS-LVO

Some studies showed the value of laboratory examination in the identification of ICAS-LVO related AIS. Dai et al. illustrated the association between serum homocysteine level (Hcy) and stroke-related multi-vascular atherosclerosis, a sign always accompany with ICAS-LVO [70]. The area under the curve (AUC) of the receiver operating characteristic curve (ROC) was 0.70 for Hcy in diagnosing multi-vascular atherosclerosis. The AUC further reached 0.87 when combining with the age of the patient. The level of Hcy can be acquired rapidly within 10 min in the laboratory, which may provide valuable assistance to the early diagnosis of ICAS-LVO. Another study by Zhang et al. pointed out the value of serum aldosterone level in diagnosing ICAS-LVO [71]. Increased serum aldosterone level was an independent predictor of ICAS and intracranial artery calcification, which can also be a potential diagnostic parameter for ICAS-LVO after being confirmed by clinical researches.

Ultrasonography, such as transcranial Doppler sonography (TCD) and transcranial color-coded Doppler sonography (TCCS), is a traditional technique for diagnosing ICAS and LAA [1, 72]. The efficacy of three-dimensional ultrasound in diagnosing cerebral vascular atherosclerosis was demonstrated to be comparable to that of CTA [73]. Clinicians are able to observe vascular stenosis from ultrasonography and speculate on the etiology of stroke. Although the popularization of CT and MRI reduced the application of TCD and TCCS in diagnosing AIS these years, the value of intravascular ultrasound (IVUS) should not be neglected [74]. IVUS is originally utilized in the diagnosis of coronary vascular diseases. The initial experience using IVUS in diagnosing AIS showed high efficiency of IVUS in identifying ICAS-LVO and vascular occlusion caused by dissection. However, IVUS is also a time-consuming operation, which cannot provide a diagnosis early enough. It might be more suitable for the etiological diagnosis of chronic stenosis.

## Discussion

In this article, current diagnostic methods and treatment strategies of ICAS-LVO were briefly reviewed, and their strengths and limitations were demonstrated. The necessity of the early and accurate diagnosis of ICAS-LVO was emphasized, and the potential methods for the early diagnosis of ICAS-LVO were summarized (Table 2).

**Table 2:** Summary of the potential methods for early diagnosis of ICAS-LVO

Number	Method	Sign	Sensitivity	Specificity	Accuracy	Reference
1	DSA	Residual stenosis after thrombectomy	Presumed 100%	Presumed 100%	Presumed 100%	Universally
2	DSA	Better collateral circulation				[17–19]
3	DSA	Truncal-type occlusion	53.1%	88.5%	84.2%	[7, 20, 21]
4	DSA	Microcatheter first-pass effect	90.9%	87.2%	88.5%	[22]
5	DSA	Tortuosity of occluded arteries				[23]
6	Histories and presentations	Ipsilateral intermittent claudication or symptoms related to TIA				[1]
7	Histories and presentations	Higher prevalence of hypertension, diabetes mellitus, dyslipidemia, smoking; the lower prevalence of atrial fibrillation				[5]
8	Histories and presentations	Lower NIHSS				[5, 24]
9	Histories and presentations	Carotid bruit				[31]
10	Histories and presentations	Brachial pulse pressure				[32]
11	Plain CT scan	High-density plaques	61.5%	68.3%	70.0%	[33]
12	Plain CT scan	Lower ASPECTS				[37, 38]
13	CTP	Smaller core infarction area				[38]
14	CTA	Poorer overall vascular condition				[42–44]
15	CTA	Better collateral circulation (grades 2-4)	33.6%	89.3%	45.9%	[45, 46]
16	CTA	Without multi-segment clot sign	32.5%	94.6%	56.2%	[47]
17	CTA	Truncal-type occlusion	53.1%	88.5%	84.2%	[21]
18	CTA	Occlusion located at proximal M1 or proximal or middle BA				[48, 49]
19	CTA	Shrinkage of artery proximal to the occlusion site				[50, 51]
20	DWI	Lower infarct volume				[39]
21	DWI	Core infarction located at deep part of the brain	93.3%	87.5%	88.5%	[40]
22	MRA	Similar to CTA				[41, 54, 55]
23	vwMRI	Decreased blood lumen area				[63]
24	vwMRI	Vessel wall enhancement				[64]
25	vwMRI	Vessel wall “white snake sign,” narrowed of both inner and outer wall, concentric enhancement, thickening of wall				[65]
26	vwMRI	Positive remodeling at the occlusion site				[67]
27	SWI	Overestimation ratio of SVS ≤ 2.003	97.1%	91.3%	93.9%	[68, 69]
28	Laboratory examination	Higher homocysteine level				[70]
29	Laboratory examination	Increased serum aldosterone level				[71]
30	Ultrasonography	Vascular stenosis				[73]
31	IVUS	Lumen stenosis				[74]

*DSA* digital subtraction angiography, *TIA* transient ischemic attacks, *NIHSS* National Institutes Health Stroke Scale, *CT* computed tomography, *ASPECTS* Alberta Stroke Program Early CT Score, *CTA* CT angiography, *BA* basilar artery, *SVS* susceptibility vessel sign

Up to now, DSA is still the golden standard in diagnosing ICAS-LVO. Besides the degree of residual stenosis after mechanical thrombectomy, indicators such as distal flow impairment, occlusion type, and “microcatheter first-pass effect” contributed to the accurate diagnosis of ICAS-LVO. However, diagnosis by DSA is based on the image after first-line treatment, which cannot provide an early diagnosis of ICAS-LVO. Clinical presentations and medical histories are the first-hand data acquired by clinicians, which can be auxiliary indicators in early diagnosing ICAS-LVO. A plain CT scan can show high-density plaques in ICAS-LVO, but it is difficult to distinguish atherosclerotic plaques from cardiogenic calcified or erythrocyte-enriched embolus. Infarct volume based on CT or CTP also shows the difference between ICAS-LVO and embolic-LVO, which deserves further confirmation. CTA can provide evidence of ICAS-LVO from different aspects, such as lumen condition of entire cerebral vessels, occlusion types and locations, and the narrowing of an artery proximal to the occlusion. However, it still cannot distinguish ICAS-LVO directly according to the characteristics of images at the occlusion site. But it still cannot distinguish ICAS-LVO directly according to the characteristics of images at the occlusion site. MRI can acquire a series of images with the diagnostic value of ICAS-LVO. DWI and MRP can distinguish ICAS-LVO by differentiating the volume and location of the core infarcted area between ICAS-LVO and embolic-LVO. MRA is able to show the lumen condition like CTA, and vwMRI can manifest vessel wall conditions, which helps better distinguish ICAS-LVO through the characteristics of the occlusion site. SVS based on SWI can help exclude the etiology of cardioembolic LVO. However, the high equipment requirement and the long scanning time limit MRI application in the case of AIS. Other methods, such as lab examination and ultrasonography, are also helpful but need further confirmation. Thus, the early diagnosis of ICAS-LVO is still a pendent issue.

**Fig. 2:**
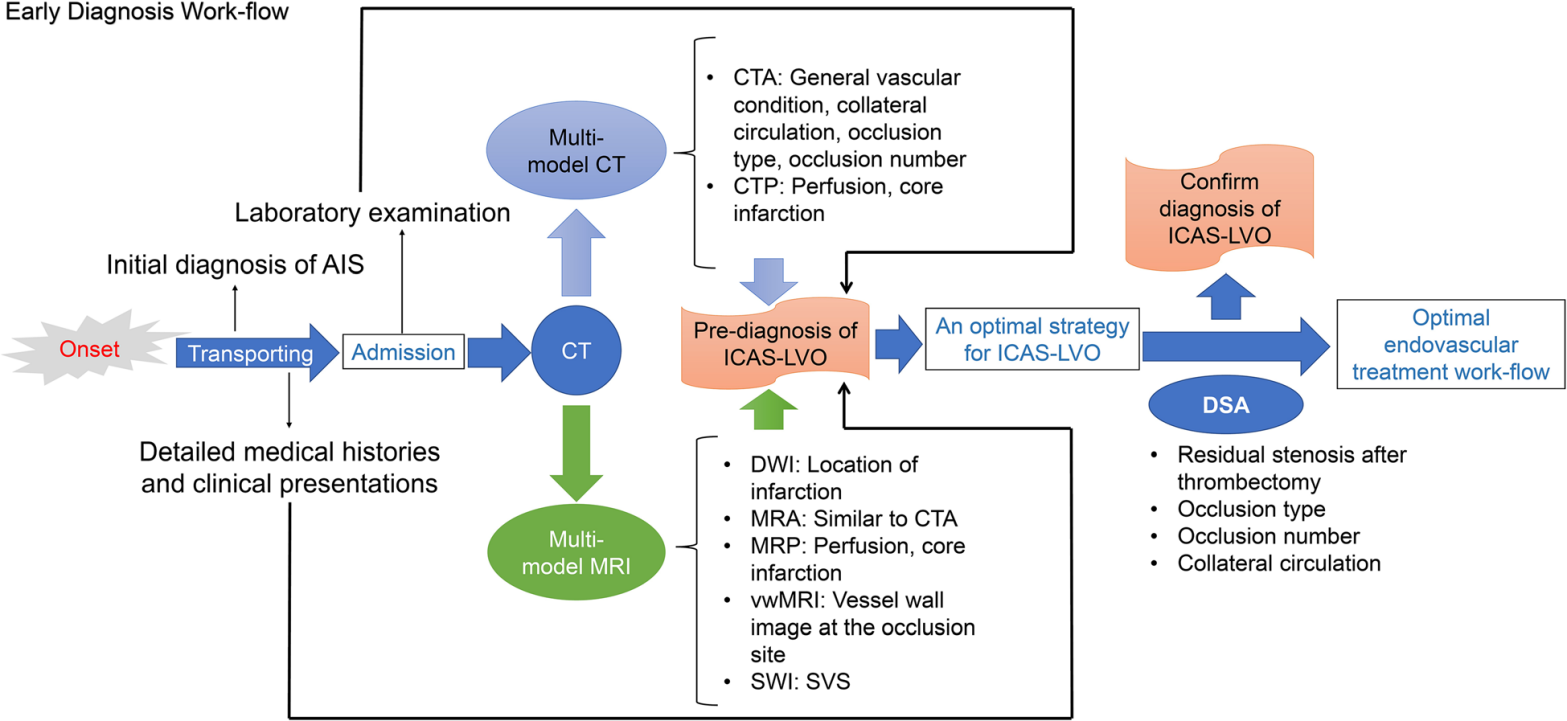
New diagnosis work-flow which might improve the efficiency of diagnosing ICAS-LVO

In our view, a comprehensive early diagnostic scale should be formulated based on the results of clinical histories, lab examinations, and the variety of signs based on multi-model CT or MRI (Fig. 2). Multi-model CT includes plain CT scan, CTP, and CTA. CTA should be an essential technique in early diagnosis because it can provide valuable luminal indicators of ICAS-LVO. Plain CT and CTP can manifest the core infarct volume, which is also a potential indicator of ICAS-LVO. Multi-model MRI, including MRI, DWI, MRP, MRA, vwMRI, and SWI, may also be a powerful method of diagnosing ICAS-LVO. It is more informative than multi-model CT, but it required much more time than multi-model CT and better hardware of the stroke center. With the evolution of MRI apparatus and reconstruction technology, the resolution will improve and the scanning time will reduce, and multi-model MRI will be a promising method in early diagnosis of ICAS-LVO in the future. Medical histories and clinical presentations are also essential evidence for ICAS-LVO. In clinical practice, these first-hand data is sometimes have not received sufficient attention, and many indicators of ICAS-LVO are ignored. The attending doctors should precisely record all symptoms and clinical histories related to the diagnosis of ICAS-LVO, such as hypertension, diabetes mellitus, dyslipidemia, and smoking.

## Conclusion

In conclusion, early and accurate diagnosis of ICAS-LVO is necessary for clinical practice and further research. Early diagnosis of ICAS-LVO should be based on medical histories, clinical presentations, and multi-model CT, which may greatly facilitate clinical practice. Other novel methods including vwMRI will be available and provide a more rapid and accurate diagnosis with the development of technology.

## Data Availability

Data sharing not applicable to this article as no datasets were generated or analyzed during the current study.

## Abbreviations

AIS: Acute ischemic stroke
ASPECTS: Alberta Stroke Program Early CT Score
AUC: Area under the curve
CT: Computed tomography
CTA: Computed tomography angiography
CTP: Computed tomography perfusion
DSA: Digital subtraction angiography
DWI: Diffusion-weighted imaging
Hcy: Homocysteine
ICAS: Intracranial atherosclerosis
IVUS: Intravascular ultrasound
LAA: Large artery atherosclerosis
LVO: Large vascular occlusion
MRI: Magnetic resonance imaging
MRA: Magnetic resonance angiography
MSC: Multi-segment clot
MT: Mechanical thrombectomy
mTICI: Modified thrombolysis in cerebral infarction
NIHSS: National Institutes of Health Stroke Scale
NR: Negative remodeling
PP: Pulse pressure
PR: Positive remodeling
ROC: Operating characteristic curve
SVS: Susceptibility vessel sign
SWI: Susceptibility-weighted imaging
TCD: Transcranial Doppler sonography
TCCS: Transcranial color-coded Doppler sonography
TIA: Transient ischemic attacks
TOAST: Trial of Org 10172 in acute stroke treatment
TOF: Time of flight
vwMRI: Vessel wall MRI
